# Editorial: Extracellular vesicles in gastrointestinal cancers

**DOI:** 10.3389/fonc.2023.1240002

**Published:** 2023-07-04

**Authors:** Liang Li, Yan Ling, Zhangjun Cheng, Norbert Hüser, William C. Cho

**Affiliations:** ^1^ Laboratory of Tumor Immunity and Metabolism, National Center for Liver Cancer, Shanghai, China; ^2^ Changzheng Hospital, Naval Medical University, Shanghai, China; ^3^ Hepato-Pancreato-biliary Center, Zhongda Hospital, School of Medicine, Southeast University, Nanjing, China; ^4^ Department of Surgery, Technical University of Munich, TUM School of Medicine, Klinikum rechts der Isar, München, Germany; ^5^ Department of Clinical Oncology, Queen Elizabeth Hospital, Hong Kong, Hong Kong SAR, China

**Keywords:** extracellular vesicles, gastrointestinal cancers, cancer diagnosis, cancer therapy, intercellular communication

This Editorial provides a synopsis of the Research Topic ‘*Extracellular Vesicles in Gastrointestinal Cancers*’. Functional macromolecules, including lipids, proteins and RNA species, are packaged in naturally nano-to-micro-sized extracellular vesicles (EVs). Interestingly, they have been found in a wide range of biofluids, including plasma, saliva, gastric acid, bile acid, amniotic fluid and urine, and are released by many different cell types (1). Gastrointestinal (GI) cancers account for more than one fourth of all cancers and are responsible for one third of cancer-related mortality worldwide (2). New diagnostic biomarkers and therapeutic options for GI cancers are urgently needed. Multiple EV biomarkers have been found and developed for use in the diagnosis, prognostic prediction and assessment of therapy outcomes in GI cancers (1). Because of their excellent biocompatibility, EVs produced from patients with cancer and laden with therapeutic and diagnostic compounds have considerable clinical utility (3).

This Research Topic aims to highlight the roles, mechanisms of action and potential applications of EVs in GI cancers. Priya et al. performed proteomic profiling of cell line-derived EVs to identify candidate circulatory biomarkers for the detection of gallbladder cancer (GBC). The authors analysed the protein content of EVs derived from the NOZ and OCUG-1 cell lines using liquid chromatography coupled to tandem mass spectrometry. The authors identified a total of 110 abundantly expressed proteins in both cell lines. The bioinformatics analysis of these proteins using the STRING tool revealed that ‘cell adhesion molecule binding’, ‘integrin binding’ and ‘cadherin binding’ are among the top molecular functions while ‘focal adhesion’ is among the top pathways associated with the EV proteins. The authors found the differential abundance of 42 proteins, including haptoglobin (HP), in GBC tissue. They reported that HP, as a representative example, was elevated in GBC patient plasma. Analysis of the receiver operating characteristic (ROC) curve for GBC versus controls revealed an area under the ROC curve (AUC) of 0.8265 for HP. This study raised the prospect that HP and other candidate proteins could be explored for potential clinical use in GBC diagnosis.

Besides, Ge et al. reviewed the recent findings regarding the role of bacteria-derived EVs in the pathogenesis and treatment of GI cancers. Outer membrane vesicles (OMVs) are a subtype of EVs produced by gram-negative bacteria. They are exosome-like particles that burst from the outer membrane and have been found to play crucial roles in a variety of bacterial life processes, including regulation of microbial interactions, promotion of pathogenesis, stress responses and biofilm formation. The authors summarised EVs of digestive tract microorganisms and their application in the treatment of digestive tract diseases. Bacterial EVs are highly immunogenic due to the presence of pathogen-associated molecular patterns (PAMPs) in bacteria, which allows them to recruit and activate immune cells at tumour locations. The anti-tumour efficacy of immunotherapy can be improved synergistically by the addition of EVs. This combination dramatically inhibits tumour development compared with monotherapy.

Cao et al. introduced tumour-derived exosomes (TDEs) and their function in various tumour immune microenvironments (TIME)-with a focus on GI cancers-and described the therapeutic strategies of exosomes in cancer immune-related therapy. Cancer cells and the immune microenvironment interact with one another. TDEs are crucial components of this equilibrium because they transport cargo from the original cancer cells and express complexes of MHC class I/II epitopes and costimulatory molecules. By analysing the specific expression and mechanism of exosomes derived from cancer cells, exosome-based targets represent a potential strategy for cancer immunotherapy. Improved strategies are needed to sort exosomes conveying genetic material. Meanwhile, enhanced extraction and detection techniques and more precise and stable strategies for modifying drugs delivered via exosomes are needed before exosome-based cancer immunotherapy could be used to treat patients.

Xie et al. reviewed the function of exosomes in GI cancers as potential drug delivery systems. Exosomes can be used as drug delivery systems and have the potential to enhance the therapeutic efficacy of cancer treatments. Exosomes as nanocarriers have the advantages of small size, negative charge, immune evasion and deep tissue penetration, making them ideal natural nanocarriers for drug delivery. Exosome-based drug delivery systems inhibit tumour proliferation and metastasis in GI cancers, have the potential to surmount drug resistance and are advantageous for immunotherapy.

Overall, this Research Topic has provided some updates on how EV-mediated intercellular communication can control the development of GI cancers or serve as a useful diagnostic biomarker. In addition, the potential of these EVs as drug delivery vehicles for the treatment of GI cancers has been highlighted, opening the door to a promising therapeutic alternative in the near future. EVs are quickly becoming an appealing tool that could eventually be used in individualised cancer diagnosis and therapy.

## Author contributions

Article writing and draft manuscript preparation: LL, YL, WC. Article modification: LL, WC, ZC, NH. All authors contributed to the article and approved the submitted version.

## References

[1] Yang P Song F Yang X Yan X Huang X Qiu Z. Exosomal MicroRNAs signature acts as efficient biomarker for non-invasive diagnosis of gallbladder carcinoma. iScience (2022) 25(9):104816. doi: 10.1016/j.isci.2022.104816 36043050 PMC9420508

[2] Huang J Lucero-Prisno DE 3rd Zhang L Xu W Wong SH Ng SC. Updated epidemiology of gastrointestinal cancers in East Asia. Nat Rev Gastroenterol Hepatol (2023) 20(5):271–87. doi: 10.1038/s41575-022-00726-3 36631716

[3] Usman WM Pham TC Kwok YY Vu LT Ma V Peng B. Efficient RNA drug delivery using red blood cell extracellular vesicles. Nat Commun (2018) 9(1):2359. doi: 10.1038/s41467-018-04791-8 29907766 PMC6004015

